# Ischemia, Immunosuppression and Infection—Tackling the Predicaments of Post-Stroke Complications

**DOI:** 10.3390/ijms17010064

**Published:** 2016-01-05

**Authors:** Raymond Shim, Connie H. Y. Wong

**Affiliations:** Centre for Inflammatory Diseases, Department of Medicine, Monash Medical Centre, Monash University, Clayton, VIC 3168, Australia; rshi20@student.monash.edu

**Keywords:** stroke, immunosuppression, infection, antibiotics, clinical outcome

## Abstract

The incidence of stroke has risen over the past decade and will continue to be one of the leading causes of death and disability worldwide. While a large portion of immediate death following stroke is due to cerebral infarction and neurological complications, the most common medical complication in stroke patients is infection. In fact, infections, such as pneumonia and urinary tract infections, greatly worsen the clinical outcome of stroke patients. Recent evidence suggests that the disrupted interplay between the central nervous system and immune system contributes to the development of infection after stroke. The suppression of systemic immunity by the nervous system is thought to protect the brain from further inflammatory insult, yet this comes at the cost of increased susceptibility to infection after stroke. To improve patient outcome, there have been attempts to lessen the stroke-associated bacterial burden through the prophylactic use of broad-spectrum antibiotics. However, preventative antibiotic treatments have been unsuccessful, and therefore have been discouraged. Additionally, with the ever-rising obstacle of antibiotic-resistance, future therapeutic options to reverse immune impairment after stroke by augmentation of host immunity may be a viable alternative option. However, cautionary steps are required to ensure that collateral ischemic damage caused by cerebral inflammation remains minimal.

## 1. Introduction

Stroke is a devastating cerebrovascular event that occurs upon interruption of blood flow to areas of the brain, due to blockage (ischemic stroke) or bursting/bleeding (hemorrhagic stroke) of a cerebral blood vessel. This resulting brain damage contributes to long-lasting disabilities and multiple functional impairments. In 2010, the global prevalence of stroke was 33 million, with an incidence rate of 16.9 million [1]. Approximately a third of strokes are fatal, which accounts for 11% of total deaths around the world, making them one of the leading causes of death and disability worldwide [1,2]. While mortality rate may be decreasing in recent times, stroke incidence is rising and predicted to increase to 23 million by 2030 [3]. Because a majority of long-term disability is due to stroke, it comes as no surprise that the global economic burden it creates on the health care system and society is immense and predicted to rise due to our ever-aging population [4,5]. In order to lower the mortality, morbidity, and social and economic strains of stroke, more effective treatments that target the underlying mechanisms of damage due to stroke are needed. While immediate cell death and brain damage is directly due to deprivation of nutrients to cells during stroke, exacerbation of neurological deficit is largely due to inflammatory processes after stroke. Additionally, despite the local inflammatory immune responses in the brain, stroke has been shown to alter systemic immunity to predispose patients to predicaments such as immune suppression and infection. A greater understanding of the pathophysiological processes that occur during and following stroke is required to shed light on potential therapeutic targets to improve patient outcomes. Thus, this review will attempt to describe the changes in local and systemic immunity after stroke, outline mechanisms of stroke-induced immune suppression and infection, and highlight potential therapeutic targets to reduce post-stroke complications and improve patient health.

## 2. Local Immune Responses and Impairment after Stroke

Stroke is an ischemia/reperfusion injury that is caused when the blood supply to the brain is insufficient. During occlusion, deprivation of oxygen and glucose can induce immediate, localized neuronal cell death at the ischemic core via excitotoxic activity [6]. Normally, glutamate is a major neurotransmitter which is released into the synapse by neurons to stimulate glutamate receptors on the postsynaptic neuron to induce depolarization via influx of calcium and sodium ions [7]. Glutamate is then internalized via active transport mechanisms that sequester glutamate action. However, the low abundance of oxygen and glucose during ischemia impairs ATP synthesis and thus clearance of glutamate does not occur. As a result, continual stimulation of glutamate receptors allows for constant neuron depolarization, generation of reactive oxygen species (ROS), and dysfunction of mitochondria to induce necrotic and apoptotic pathways (reviewed in [6,8]). 

Subsequent release of damage associated molecular patterns (DAMPS) by dying cells activates resident microglial cells through stimulation of pathogen recognition receptors (PRRs), such as toll-like receptor (TLR) 2 and TLR 4 [9,10,11]. This triggers downstream inflammatory signaling cascades—inducing the activation of NF-κB and mitogen-activated protein (MAP) kinase pathways. Within minutes, local production of pro-inflammatory molecules occurs at the site of occlusion [12]. Such molecules include cytokines, such as interleukin-(IL-) 1β, IL-6 and tumor necrosis factor-(TNF-)α [13,14,15], chemokines and their receptors [16,17], integrins and adhesion molecules, such as Very Late Antigen-4 (VLA-4), Intercellular Adhesion Molecule-1 (ICAM-1) and Vascular Cell Adhesion Molecule-1 (VCAM-1) [18]. 

Upon resolution of the vascular blockage, re-oxygenation of the brain promotes mitochondrial-mediated production of ROS to cause further injury to cellular components and promote cell death in surrounding neurons, glial cells and blood vessels [19]. The production of the aforementioned cytokines, chemokines, adhesion molecules and ROS are all pro-inflammatory and triggers the recruitment of peripheral immune cells in an attempt to initiate clearance of cell debris and healing in the brain. However, excessive infiltration of leukocytes is found to be more harmful. As the brain is encased within the skull, swelling, as a result of infiltrating immune cells, can increase the intracranial pressure. High intracranial pressure has been shown to increase fatality by up to 80% in stroke patients, and reducing the intracranial pressure in patients within 48 h after stroke can reduce death and improve stroke outcomes [20,21].

Various immune cells have different roles in infarct development after stroke. Macrophages and neutrophils have been found to infiltrate within 24 h following reperfusion [22]. Macrophages are amongst the first to enter the brain during reperfusion and their role in infarct development is under debate [22]. While some studies show that macrophages can have neuroprotective effects, others suggest they can be neurodegenerative. Bone marrow-derived macrophages have been reported to produce Transforming Growth Factor Beta (TGF-β) and confer neuroprotection following stroke by initiating healing and clearance of cell debris [23,24]. In contrast, macrophages are also a major source of IL-1β, TNF-α and ROS, adding to further inflammatory insult after stroke [25,26]. The controversial findings of macrophages in infarct development may be explained by classical or alternative activation of macrophages, whereby classically activated macrophages (M1 macrophages) exacerbate damage while alternatively activated macrophages (M2 macrophages) assist in repair and neurogenesis [23]. In fact, macrophages in the brain of post-stroke mice initially express M2 markers; however, a shift toward an M1 phenotype occurred following 5 days after ischemia which may result in the release of inflammatory mediators to exacerbate damage in the brain [27].

Neutrophils are also known to rapidly infiltrate ischemic tissue within hours after stroke. While the role of neutrophils is controversial, trends in literature suggest that neutrophils contribute more toward detrimental outcomes in the brain following stroke [18]. Preventing the migration of neutrophils into cerebral ischemic tissue could reduce infarct volume and improve neurological outcomes in mice [22,28,29,30,31]. However, other recent studies do not support the damaging role of neutrophils as depletion of neutrophils did not reduce brain damage after stroke [32]. Interestingly, similar to macrophages, while an N1 phenotype in neutrophils was observed in the brain of post-stroke mice, therapeutic activation of the peroxisome proliferator-activated receptor-γ could polarize neutrophils toward an N2 phenotype to assist in neuroprotection by clearance of neutrophils and resolution of inflammation [33].

Influx of lymphocytes usually occurs after 2–3 days following stroke. Most notably, T cells have been found to contribute to infarct development through production of IL-1β, interferon gamma (IFN-γ) and macrophage inflammatory protein (MIP)-2 [34,35]. Mice deficient in T and B cells had similar infarct sizes compared to wild-type mice, whereas reintroduction of T lymphocytes increased infarct sizes post-stroke [36]. In contrast, B cells may be neuroprotective as B cell deficient mice tended to have increased infarct volume, suggesting an immune regulatory role of B cells in the brain after stroke, possibly in an IL-10 directed manner [37,38]. The somewhat controversial roles of various immune cells in infarct development are complex and have been more extensively reviewed elsewhere [39]. Interestingly, recent research into the role of the regulatory T cells (Tregs) after stroke have revealed their contentious roles in infarct development. On one hand, Treg depletion using CD25-specific monoclonal antibodies resulted in exacerbated long-term brain damage and worsened functional outcome in experimental stroke, potentially due to the lack of IL-10 production [40]. On the other hand, Treg depletion by diphtheria toxin in Foxp3^DTR^ (diphtheria toxin receptor) mice did not alter ischemic lesion volume 3 days after stroke to argue against the neuroprotective role of Tregs [38]. Confoundingly, pre-stroke depletion of Tregs by diphtheria toxin actually reduced infarct development and functional outcomes within 24 h following transient mid-cerebral artery occlusion (tMCAO) model [41]. In fact, it was observed that Tregs disrupt microvascular function in the brain via lymphocyte function-associated antigen 1 (LFA-1)/ICAM-1 action to worsen brain damage [41]. As the role of Tregs remains under dispute, it is therefore vital that the controversial role of Tregs in infarct development is elucidated before translating findings into stroke patients. There are other promising neuroprotective therapies that aim to reduce inflammation by exploiting activation of suppressor cells and preventing immune cell recruitment by blocking adhesion molecule action in experimental models [27,29,33]. However, as of yet, these have not been effective in humans [42]. In contrast, drugs currently in use to treat multiple sclerosis, including natalizumab and fingolimod, and may have potential in attenuating infarct development [43]. Natalizumab is a monoclonal antibody that blocks α4-integrin (a subunit of VLA-4) to prevent the infiltration of lymphocytes into the brain [18]. The efficacy of natalizumab is currently under dispute as experimental studies have shown either a neuroprotective [18] or benign [44] effect of natalizumab in reducing brain damage. Despite this, a preclinical phase III trial is in progress and may shed light on the effectiveness of natalizumab in improving stroke outcomes [45]. Similarly, fingolimod is an agonist of the sphingosine-1-phosphate (S1P) receptors, S1P1, S1P3, S1P4 and S1P5, which limits the migration of immune cells out of lymph nodes and into the central nervous system (CNS) [46]. Promisingly, fingolimod treatment after stroke has been found to effectively reduce brain infarct development in both experimental [47,48] and clinical stroke [49].

The excess infiltration of leukocytes can be largely explained by increased permeability of the blood brain barrier (BBB) [50]. Normally, the immune privileged status of the brain is regulated by the BBB; however, disruption of BBB integrity can allow for unregulated entry of immune cells [51,52]. Many molecules that are released after stroke, such as ROS, can increase the permeability of the BBB and disrupt its homeostasis [51,52]. In addition, uncontrolled macromolecule infiltration can cause movement of fluid into the brain, resulting in edema [51,52]. Furthermore, permeabilization of the BBB also induces the exposure of CNS antigens to the periphery, potentially leading to acquired autoimmunity [53]. Notably, loss of natural killer (NK) cell tolerance to cell bodies and axons was shown to promote lesion development in post-stroke mice [54]. Additionally, immune responses against myelin basic protein (MBP) and glial fibrillary acidic protein (GFAP) have been associated with larger cerebral infarctions and increased the likelihood of worse outcome in stroke patients [53,55]. Therefore, stroke disrupts the normally tightly regulated homeostasis of the immune system and result in further collateral brain damage following the initial ischemia event. However, stroke also has systemic repercussions that leave patients in a worsened state of health. 

## 3. Infections after Stroke

There is no doubt that sequences of highly inflammatory events add to cerebral insult after stroke and leads to detrimental outcomes, however, despite all the neurological damage that occurs after stroke, a major clinical complication that stroke survivors encounter is infection. It has been reported that between 23% and 65% of patients acquire infection after stroke, with pneumonia and urinary tract infections (UTI) being most common [56,57]. Infections contribute to worsening of clinical outcomes, increased risk of recurrent stroke and death [58,59]. In fact, stroke-associated infections can account for approximately 30% of fatality in stroke patients [20,60,61]. As a result of an immune response to post-stroke infection, levels of inflammatory molecules such as IL-6 and C-reactive protein are elevated and are associated with poor patient outcomes [62,63]. 

Diagnosis of infection after stroke can be challenging as the criteria of diagnosis are inconsistent between studies. Hallmark signs of infection in clinics, such as fever and inflammation, can be presented by patients as a consequence of neurological damage that disrupts homeostatic regulation of body temperature [64]. On the other hand, use of aspirin and paracetamol can veil infection by reducing fever. Other symptoms, such as delirium, neurological deterioration and dehydration, are indicative but are not specific for infection [65]. Chest radiographs are largely relied upon, though with only a 65% sensitivity. Additionally, the detection of pneumonia with this method may only be useful in later stages of infection due to a lack of infiltrates [66]. Thus, there is a need for improved and universal diagnostic methods and markers to more accurately identify infections after stroke. Interestingly, magnetic resonance imaging (MRI) was used to measure severity of lung infection in post-stroke mice [67]. This may be a potential translational opportunity to rapidly detect and diagnose chest infection in a clinical setting.

Identifying whether patients will succumb to infection could allow for early interventions to reduce post-stroke bacterial burden and improve clinical outcome, however it can be difficult to predict. One study identified a range of independent risk factors that could predict pneumonia after stroke with 76% sensitivity and 88% specificity [59]. These factors included dysphagia, National Institute of Health Stroke Scale (NIHSS) of ≥10 and non-lacunar basal-ganglia infarction [68]. Similarly, a 12-point scoring system was devised to predict stroke-associated pneumonia based on commonly tested variables such as age, blood pressure and leukocyte levels, which had 77.6% sensitivity and 84% specificity [69]. Loss of lymphocytes may also be a predictor of infection after stroke. Indeed, while leukocyte numbers increase, lymphopenia occurs within 6 h and last for at least 6 days after stroke in patients [70]. Consequently, stroke patients that exhibit signs of lymphopenia also acquired infections strongly emphasizing the critical role of lymphocytes in host bacterial defense after stroke. In addition, decreased TNF-α production and reduced HLA-DR expression in monocytes were reported and may be viable predictive markers to infection [70,71]. However, similar attributes in stroke patients without infection were also found and this puts the reliability of these markers into question when it comes to predicting infection onset after stroke. Similar findings were recently reported, where total leukocyte counts were increased in the infected cohort of stroke patients [72]. Intriguingly, recovery of circulating lymphocyte numbers was seen one day after stroke in non-infected patients, whereas infected patients demonstrated persistent lymphopenia [72]. These findings further support the notion of lymphopenia as a predictor of post-stroke infection, and future therapeutics may require boosting lymphocyte levels in order to reduce infection. Importantly, though, immunological changes seen in these studies and others allude to the phenomenon that is stroke-induced immune suppression. 

## 4. Stroke-Induced Immune Suppression

Although initial systemic inflammation, characterized by circulating IL-6 and IFN-γ production, may peak within 6 h following stroke [73], it has become increasingly evident that systemic immune suppression takes place as a compensatory mechanism against brain damage [74]. As previously discussed, despite an increase in granulocyte numbers, infection still occurs in patients within days after stroke [70,72]. This may suggest impairments in innate immunity contribute to infection development in the acute stages after stroke, however this has not been as comprehensively explored. Neutrophils are well-known to rapidly respond and migrate to sites of infections where they elicit protection against pathogens via expression of soluble inflammatory mediators and superoxide generation [75]. Patients with hemorrhagic stroke had impaired neutrophil respiratory burst, which suggests that future therapeutics may target immunomodulatory pathways with an aim to restore neutrophil function [76]. This coincides with a recent study that also reported impairment of respiratory burst by circulating granulocytes and monocytes in ischemic stroke patients. Interestingly, the stroke-induced neutrophil impairment was attributed by the granulocyte and monocytes responses to catecholamines [77].

Monocytes and macrophages are phagocytes that can engulf pathogens and infected cells to alert and prime the rest of the immune system [78]. Splenic macrophages/monocytes have been shown to lack VLA-4 which prevented the migration of these cells into other tissues [79]. Most importantly, macrophages have a large role in bridging the innate and adaptive immunity by interacting with T cells to initiate specific immune responses [80]. In order to trigger a sustained, specific and effective immune response against a pathogen, antigen presenting cells (APCs), like the macrophage, must recognize and present foreign peptide antigens through major histocompatibility complexes (MHC) and present costimulatory molecules to T lymphocytes [81,82]. This, in turn, initiates other parts of the immune system for an anti-pathogen response. Previous studies have shown that expression of MHC class II (or HLA-DR in humans) is reduced [72,83], costimulation is less efficient [84], and production of pro-inflammatory cytokines by monocytes is decreased after stroke [71]. Collectively, these studies suggest that stroke interferes with the normal functions of the immune system to the extent that the bactericidal immunity is impaired to leave the host susceptible to infections.

Another indicator of immune suppression is the macroscopic shrinking of immune organs, shown to occur within 12 h following stroke in mice [85]. Most notably, size reduction of spleen and thymus may be partly due to apoptotic death of splenocytes [85,86]. The stroke-induced death of splenocytes resulted in reduced production of T cell mitogenic factors and thus prevents T cell proliferation and inflammatory cytokine production [79,85]. Additionally, migration of cells out of the spleen and into the parenchyma in the brain may also contribute to the reduction of spleen size [86]. Moreover, induction of Tregs and loss of B cells in the spleen was thought to further compromise host pathogen defenses [79]. 

Adaptive immune changes can largely predispose to chronic susceptibility to infection [87]. Particularly, in T cells, a shift of from a cell-mediated inflammatory T-helper 1 (T_H_1) type response to a humoral-mediated anti-inflammatory T-helper 2 (T_H_2) type response is suggested to occur to protect the brain from further inflammatory damage and promote tissue repair and neuronal regeneration [88,89]. However, this shift occurs to the extent that the host’s systemic immune system is suppressed and becomes ineffective in fighting pathogens, resulting in increased susceptibility to infections. The systemic cytokine milieu plays an important role in determining immune suppression and the type of T-helper response that predominates. The production of immunosuppressive and T_H_2-associated cytokines mediates the immune shift away from a T_H_1 response and toward a T_H_2 response after stroke. In particular, IL-10 secretion by monocytes, dendritic cells, and Tregs is greatly increased after stroke in both mice and humans [79,90], and can act upon many immune cell types to avert from a pro-inflammatory response. The production of IFN-γ by T_H_1 cells, proliferation of T cells and cytokine responses, TNF-α production by macrophages and cytokine production by monocytes are all inhibited by IL-10 to further emphasize its role in the immune shift toward a T_H_2 response and immune suppression after stroke [91]. Decreased levels of serum IFN-γ and TNF-α, well known T_H_1 cytokines, were seen 12 h after stroke in mice [85]. In fact, reduced serum IFN-γ level is critical for stroke-induced susceptibility to infection as adoptive transfer of IFN-γ-producing splenocytes resulted in a significant reduction of bacteria in the blood and lungs after stroke [85]. 

Although immune suppression is apparent following stroke, the mechanism in which this occurs is not well understood. One explanation is that an early, sustained inflammatory response may exhaust the immune system and ultimately lead to immune suppression [79,92]. Another explanation may be that the brain attempts to suppress the immune system in order to prevent further cerebral inflammation [79]. Indeed, neural control of immunity, possibly by the hypothalamic-pituitary-adrenal (HPA) axis and sympathetic nervous system (SNS), is an emerging concept and is thought to play the biggest role in the systemic immune shift after stroke. Briefly, the HPA axis is the collaboration of the central nervous system (CNS) and endocrine system that regulate bodily functions whereby corticotrophin releasing hormone (CRH) is released by the hypothalamus in response to stress [93]. CRH then stimulate the production of adrenocorticotropic hormone (ACTH) by the anterior pituitary gland which can then travel to the adrenal gland to induce glucocorticoid production as an end product [94]. Production of IL-1, IL-6, IL-10 and TNF-α as a consequence of cerebral insult from stroke can be sensed by the hypothalamus to activate the HPA axis for excessive glucocorticoid release [92]. Similarly, stroke-induced activation of the SNS results in the secretion of catecholamines by the adrenal medulla and nerve terminal [94]. Immune cells express receptors that can be stimulated by glucocorticoids and catecholamines to promote an immune suppressive response [95].

**Figure 1:**
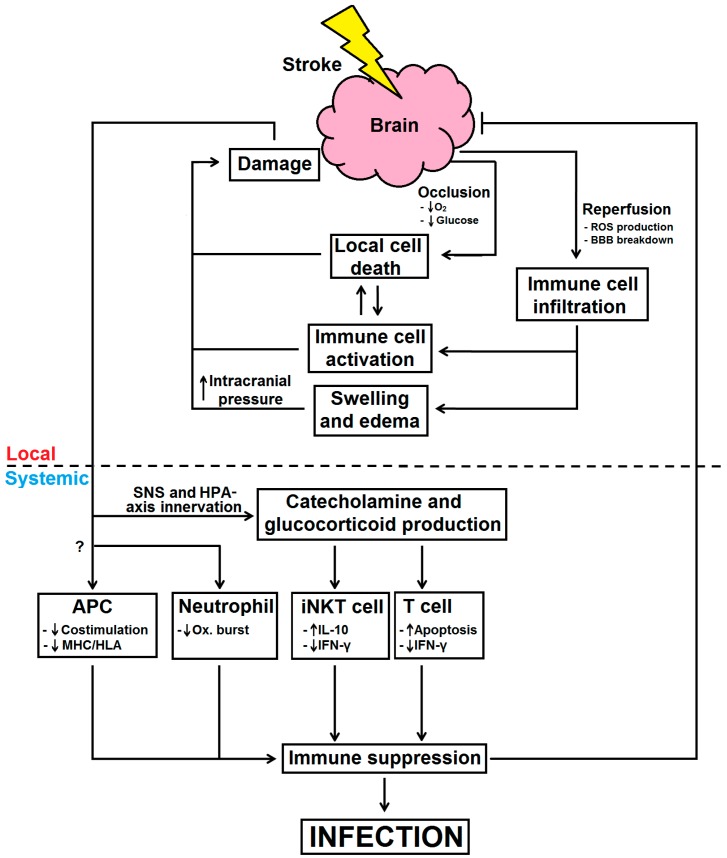
Immunological processes and mechanisms for infection after stroke. During a stroke, interrupted blood flow into the brain deprive cells of vital nutrients and thus undergo cell death. This in turn activates local immune cells to produce pro-inflammatory factors, which can have deleterious effects on the brain. Additionally, restoration of blood flow into the brain allows for reperfusion, resulting in ROS production and BBB breakdown. This allows for excessive recruitment and infiltration of circulating immune cells into the brain to promote swelling and increased intracranial pressure to exacerbate brain damage. In an attempt to reduce damage and resolve local inflammation, the brain responds by activating the SNS and HPA-axis in order to produce catecholamines and glucocorticoids. Catecholamines and glucocorticoids can act upon iNKT cells and T cells, respectively. Meanwhile, APC and neutrophils are also impaired to the paint where their bactericidal activities are insufficient, all of which resulting in systemic immune suppression and therefore leaving stroke sufferers more susceptible to infections. The brain and stroke are represented by a cloud and a bolt, respectively. Arrows denote “activate” or “induce”; blunted arrows denote “inhibit” or “prevent”. Dashed lines denote activities locally in the brain versus systemically in the periphery.

Catecholamines signaling through β-adrenergic receptors on immune cells have been shown to reduce TNF-α while promoting IL-10 production. Stimulation of β-adrenergic receptors can also inhibit cytotoxic T-lymphocyte-associated protein (CTLA)-4 expression on T cells [96]. On the other hand, glucocorticoids can act upon T cells, to reduce IFN-γ production and induce apoptotic death, and monocytes to promote IL-10 secretion [85]. However, administration of propranolol, a β-adrenergic receptor blocker, but not RU486, a glucocorticoid receptor blocker, into post-stroke mice have been shown to reduce bacterial complications and mortality after stroke, strongly suggesting a role catecholamines in inducing immune suppression post-stroke [85]. Additionally, the apoptotic effect of catecholamine and glucocorticoid release after stroke may account for the lymphopenia observed following stroke [85]. In fact, the concentration of catecholamines in plasma has been shown to correlate with the amount of post-stroke immune suppression in humans. A recent study in our laboratory found that hepatic invariant natural killer (iNKT) cells are in a prime position to detect and respond to distant brain damage in a murine model of stroke [97]. In that study, we showed that the behavior of iNKT cells are impaired by β-adrenergic signaling after stroke [97]. Furthermore, blocking of β-adrenergic receptors with propranolol resulted in increased IFN-γ production and reduced the bacterial burden in post-stroke mice [97]. However, this protection conferred by propranolol administration was not seen in mice deficient of iNKT cell, demonstrating the role of iNKT cells in catecholamine-mediated immune modulation after stroke. Others have also investigated the impact of catecholamines and glucocorticoids on immune suppression after stroke [98] but overall, these mechanisms work together to dampen the inflammatory immune responses to minimize further immune pathology in the brain, although this comes at the cost of susceptibility to infections in the host [97] (Figure 1).

## 5. Other Causes of Infection after Stroke

Pneumonia and UTI’s are the main types of infection after stroke. Pneumonia is seen to be the most common with an incidence rate of up to 57% [99], while UTIs can occur between 11% and 27% of stroke patients [100]. The occurrence of stroke-associated pneumonia has been shown to worsen clinical and neurological outcomes, and therefore studies have recently investigated the mechanisms in acquiring pneumonia after stroke. The most common cause for stroke-associated pneumonia was thought to be solely aspiration and dysphagia that result from impaired swallowing function [101]. Defects in the swallowing mechanisms may be partially explained by depletion of substance P following stroke [102]. Substance P has roles in the coughing and swallowing reflex. After stroke, dopamine production by the corpus nigrostriate is reduced and thus substance P secretion by the glossopharyngeal nerve is downregulated [101]. This promotes aspiration by allowing entry of gastric contents into the lungs to result in pneumonia [103]. In addition, while the lack of substance P has been shown to enhance aspiration and dysphagia following stroke, the pathophysiology of aspiration and dysphagia should be noted to be complex and involve a variety of aspects, including decreased level of consciousness, body positioning in bed, mechanical ventilation, and patient immobility [104]. However, it is now known that aspiration and dysphagia only partially explain the high frequency and susceptibility of stroke patients to pneumonia. There is evidence in experimental and clinical studies that show that aspiration is not the only cause of pneumonia after stroke. In fact, intranasal administration of only 200 colony-forming units (CFU) of *Streptococcus pneumoniae* was necessary to cause pneumonia and bacteremia in post-stroke mice, whereas sham-surgery mice needed 1000-fold greater amount of bacteria for a similar severity of pneumonia [105]. In a clinical setting, the incidence of pneumonia in stroke patients who were fed via gastric tube to prevent aspiration was 44% to further suggest alternative mechanisms in pneumonia susceptibility [106]. Nonetheless, later on it was found that gastric tube feeding could predispose to infection [107]. However, aspiration has been shown to occur in healthy individuals at a similar extent as in stroke patients, though pneumonia does not develop [108]. Stroke-induced immunosuppression is now more universally acknowledged to be the main explanation for susceptibility to infection after stroke, with aspiration and dysphagia being a contributor rather than the sole cause. 

While infections are common after stroke, the causative agent remains elusive and may be contributed by a number of pathogens. Types of bacteria that have been commonly found in the sputum and urine of stroke patients include *Streptococcus pneumoniae*, *Staphylococcus aureus*, *Klebsiella pneumoniae* and *Psuedomonas aeruginosa* [109]. Surprisingly, reports of common gut bacteria have been reported with *Escherichia coli* and *Enterobacter cloacae* being two of the most frequently detected [110]. Indeed, more than 95% of bacterial cultures of blood and lungs of stroke mice were found to be made up of *Escherichia coli* [85]. However, a number of studies could not identify the causative agent of infection in a large portion of their stroke patient cohorts [68,107,111]. Reasons for this may be due to low yields of sputum/aspirate, as neurological deficit makes it difficult for patients to produce samples, and challenges in culturing the causative agent as some microbes require extremely specific culture conditions to grow [112]. Overall, understanding or identifying the causative agents of infection can allow for more targeted treatments to reduce post-stroke bacterial complications.

It may appear that we have evolved to react to stroke injury with an apparently maladaptive response (post-stroke infection). During stroke, self-epitopes, which are normally shielded from the systemic immune system by a number of mechanisms, may become exposed to adaptive immunity. This may ‘educate’ the immune system to react to self-antigens in the CNS, and, ultimately, lead to autoimmunity or autoaggression. According to this concept, by globally down-regulating innate and adaptive immunity, stroke-induced immune suppression may help to prevent post-injury autoimmunity. Indeed, brain-specific antigens can be measured in the blood plasma after stroke [113]. At present, there is no epidemiological evidence that patients with stroke have an increased incidence of autoimmune CNS disorders (such as multiple sclerosis). This might point to effective control of autoimmunity under injury conditions, which would come at the price of an increased susceptibility to infection. Interestingly, infections after stroke have been shown recently to predispose patients to autoimmunity against brain antigens [114]. Systemic inflammation as a response to infection is thought to induce an immune response against infection-associated antigens, but immune responses against self-antigens may occur as a collateral result. To mimic post-stroke infections in an experimental model, post-stroke rats, administered with lipopolysaccharide (LPS) to induce a systemic inflammatory response, had a greater T_H_1-mediated immune response toward MBP [115] and increased mortality rates [116]. Additionally, immune responses against MBP and GFAP were found to be more common in stroke patients with acquired infection and had worsened outcomes compared to stroke patients without infection [53]. This may explain why infection is independently associated with poor outcomes and therefore emphasises the importance in reducing the incidence of infections after stroke.

## 6. Treatments for Stroke-Associated Infections

### 6.1. Antibiotics

The obvious strategy and current “gold-standard” treatment to combat infections is the use of antibiotics upon diagnosis of infection [67]. However, treatment at this stage could decrease the rate of recovery, worsens the functional outcome of patients, increases the likelihood of recurring stroke, and extends the amount of suffering for the patient [117]. A main focus in recent years has been prophylactic administration of broad-spectrum antibiotics to avoid the clinical problems that are associated with infections. Experimentally, post-stroke mice that were on preventive antibiotic treatment (PAT) with fluoroquinolones had improved survival rates and neurological outcomes compared to mice either with antibiotic treatment upon diagnosis of infection or without antibiotic treatment at all [67,118]. However, clinical studies on the effectiveness of PAT are controversial and not practical. In fact, a number of studies do not support this strategy to reduce infection after stroke [88,107,119,120].

A follow-up study with moxifloxacin, a fourth generation fluoroquinolone, was conducted in the Preventive Antibiotic Therapy in Ischemic Stroke (PANTHERIS) clinical study [108]. With per-protocol (PP) analysis (*n* = 66), it was found that prophylactic treatment with moxifloxacin reduced infections by 24.8% (*p* = 0.032), though this treatment did not seem to be beneficial using with intention-to-treat (ITT) analysis (*n* = 79; *p* = 0.114), which included all recruited patients. ITT analysis may be more viable to evaluate the effectiveness of this treatment as excluding some patients for PP analysis may dramatically impact results due to the small group size in this study (*n* = 31 in placebo group and *n* = 35 in treatment group). Overall, the main finding in the PANTHERIS study was that patients did not have improved clinical outcomes or reduced mortality after 6 months despite reduced infection rates and thus discourages prophylactic use of moxifloxacin as a preferential treatment over current treatment guidelines to reduce post-stroke infection.

Similar results were found for the most recent studies that evaluated the effectiveness of PAT. The preventive antibiotics in stroke study (PASS) used ceftriaxone, a third generation cephalosporin, to determine whether treatment could reduce infection to improve functional outcomes after stroke [121]. Previous studies have shown that ceftriaxone had neuroprotective qualities by reducing excessive excitatory mechanisms and thus has potential to reduce neurological deficit after stroke [7,122]. Patients were administered ceftriaxone within 24 h after onset of stroke symptoms, which was continued once daily for 4 days. Functional outcomes, defined by the modified Rankin Scale (mRS), were assessed at 3 months. The main finding was that infections could be reduced after ceftriaxone treatment, although improvement of neurological and functional outcome was not observed. Interestingly, the study found that prophylactic treatment with ceftriaxone could significantly reduce rate of UTI but not of pneumonia between the treatment group and the placebo group. Despite this, it should be noted the proportion of patients with infection in this study was relatively low, whereby study populations of previous trials were often limited to patients with severe strokes. In the PASS study, patients with mild strokes were included, and the median NIHSS was 5. The fact that functional outcome does not improve despite absence of infection is perplexing as multiple studies have shown the association of infection and worsened outcomes [61,111,123]. However, there may be some explanations for this. One explanation may be that infection may merely be marker or a symptom of the extent of poor functional outcome. This may be feasible as other studies that showed prophylactic antibiotic interventions could reduce infection rates but not improve functional outcomes; however, this requires further investigation for confirmation [112,123,124]. Another explanation stems from the finding that ceftriaxone did not significantly reduce incidence of pneumonia, which suggests that pneumonia is the greater cause for worsened outcomes after stroke. Indeed, this would explain why, despite the decreased infection rates, the functional outcomes of the stroke patients did not improve [121].

In the STROKE-INF study, prophylactic use of clarithromycin in combination with either amoxicillin or co-amoxiclav was given within 48 h after stroke for 7 days in dysphagic patients where occurrence of pneumonia and patient functional outcomes at 90 days was assessed [111,125]. Interestingly, treatment did not reduce the incidence of pneumonia or mortality compared to the untreated group and actually lengthened time of the patients’ hospital stay. Again, these studies further suggest prophylactic antibiotic treatment is not better than standard treatments. 

Lastly, another possible reason that antibiotic treatment is not effective is the unsuitable use of particular antibiotics due to antibiotic resistant bacteria. One study took sputum samples from patients with stroke-associated pneumonia, cultured for bacteria and tested their drug sensitivity against a range common antibiotics including penicillin, tetracycline, ceftriaxone and moxifloxacin [109]. The prevalent strains of bacteria that were found in the sputum were *Psuedomonas aeruginosa* (23.92%), *Staphylococcus aureus* (15.32%) and *Escherichia coli* (14.25%). The study found that drug resistance against moxifloxacin, used in the PANTHERIS trial [108], was 52.63% in *S. aureus* [109]. Strikingly, resistance rates against ceftriaxone, the antibiotic of choice used in the PASS trial [107], was 100% for both *Escherichia coli* and *Psuedomonas aeruginosa* [109] which may offer an explanation to why pneumonia was not reduced in ceftriaxone-treated stroke patients. Importantly, resistance rates will differ between hospitals and therefore future studies should assess for antibiotic resistance in the patient cohort. Indeed, infections should be prevented by using selective antibiotics that take into consideration the results of antibiotic testing [109]. However, the rise in antibiotic resistant pathogens implies that it is high time for antibiotic-independent therapies to more effectively reduce infections after stroke.

### 6.2. Immune Modulation

The impact that stroke has on the immune system is complex and, as discussed, innervation of the sympathetic nervous system and HPA axis can result in an immune shift towards a T_H_2 response after stroke to leave the host susceptible to infections. Targeting these pathways to prevent the immune shift following stroke may offer a viable alternative to antibiotics to reduce infections. Catecholamines are produced as a result of cerebral ischemia and can act upon β-adrenoreceptors on immune cells to induce apoptosis and immune suppressive pathways. Therapeutic blocking of the β-adrenergic receptor after stroke has been shown to reduce infections in experimental models of stroke by counteracting the immunosuppressive properties of catecholamines [85,97]. Intriguingly, the occurrence of pneumonia in post-stroke patients that were on β-blockers, prior to ictus and during hospitalization, was less than that of patients who did not receive β-blockers. In addition, β-blocker therapy significantly reduced 30-day mortality in stroke patients [126]. A recent study that examined 5212 stroke patients from a Virtual International Stroke Trials Archive also found that on-stroke β-blocker therapy was associated with reduced mortality and that both pre-stroke and on-stroke treatment with β-blocker reduced pneumonia frequency [127]. In contrast, the most recent clinical trial saw that, while using β-blocker treatment reduced the occurrence of UTI, it did not provide adequate protection against pneumonia and the treatment group actually had a greater mortality after 30 days compared to patients not on β-blockers [128]. Taken together, it is clear that the use of β-blocker therapy in post-stroke patients requires further study in order to elucidate its effectiveness in reducing stroke-associated infections. 

We recently showed that the stroke impairs iNKT cell function within hours after stroke to mediate immune suppressive pathways and result in increased host susceptibility to infections in an experimental setting [97]. Interestingly, specific activation of iNKT cells with α-galactosylceramide (α-GalCer), a glycolipid that is presented via CD1d, could significantly increase systemic IFN-γ production and reduce bacteria in all of the tissue examined post-stroke, while brain damage was not exacerbated. This study showed that iNKT cell is one of the major mediators of the immune shift after stroke and modulating their responses using α-GalCer was efficient in reducing infections after stroke [97]. This merits further study in the design of novel α-GalCer analogues that can skew or bias iNKT cell responses in altering host immunity after stroke to reduce infectious complications.

Additional studies in the mechanisms of brain-immune system axis are required to reveal potential alternative therapeutic options. However, cautionary steps should be taken with immunomodulatory treatments. Suppression of the immune system after stroke is thought to be a brain-protective mechanism as the majority of penumbral damage is governed by inflammatory events [14,89]. Therefore, boosting the immune system with immunomodulatory therapy to reduce stroke-associated infection may promote further collateral damage in the brain. An optimal immunomodulatory regime should reduce infection burden without exacerbating brain damage. Future research may also choose to consider a combination therapy to minimize immediate cerebral insult and prevent infections during the post-acute phrase of stroke for better patient outcomes.

## 7. Conclusions

Damage to the brain after stroke is now known to be a largely immune mediated event. As a compensatory response to the highly inflamed environment within the skull, the activation of the sympathetic nervous system and hypothalamic-pituitary-adrenal axis results in the production immune suppressive molecules including catecholamines and glucocorticoids, respectively. The brain-immunity axis is a recently identified and complex pathway that induces immune suppression after stroke. This is believed to be a defensive mechanism of the brain with an attempt to lessen further cerebral damage, however rendering the host more susceptible to infections. Infections such as pneumonia and UTI are extremely common in stroke patients and have been associated with poorer functional outcomes and increased mortality. Therefore, a recent focus has been to reduce infection to improve patient health. Much attention has been drawn to preventive antibiotic treatment, though the majority of clinical studies have swayed against prophylactic antibiotic administration due to a failure in reducing pneumonia and improving clinical outcomes. Importantly, recent studies reveal an underlying issue in the inconsistency of pneumonia diagnosis across clinics. Additionally, with the growing emergence of antibiotic-resistant pathogens, alternative therapies to reduce infection are required. Immunomodulation therapies that augment the immune system to combat infections is an attractive avenue, however care is needed to ensure further cerebral injury is avoided. Future studies may want to focus on the precise mechanisms of immune suppression after stroke in order to unveil alternative therapeutic targets to reduce stroke-associated infections.
